# Fracture risk assessment in home care patients using the FRAX^®^ tool

**DOI:** 10.1590/S1679-45082018AO4236

**Published:** 2018-09-09

**Authors:** Vitor Moraes Rocha, Heloisa Amaral Gaspar, Claudio Flauzino de Oliveira

**Affiliations:** 1 Home Doctor, São Paulo, SP, Brazil.; 2 Hospital Israelita Albert Einstein, São Paulo, SP, Brazil.

**Keywords:** Home nursing, Aged, Health of the elderly, Fractures, bone, Femoral fractures, Hip fractures, Patient safety, Assistência domiciliar, Idoso, Saúde do idoso, Fraturas ósseas, Fraturas do fêmur, Fraturas do quadril, Segurança do paciente

## Abstract

**Objective:**

To assess the ten-year risk of hip and osteoporotic fracture in home care patients using the FRAX^®^ tool.

**Methods:**

A retrospective, cross-sectional observational study including patients aged ≥ 40 and ≤ 90 years and receiving home care from a private provider. The risk of fracture was calculated using an online calculator. High risk was defined as risk of hip fracture greater than 3% or risk of osteoporotic fracture greater than 20%. Data were expressed as absolute number (n), relative frequency (%), mean, standard deviation (±) and probability value (p).

**Results:**

Eighty-three (37.7%) out of 222 patients were at high risk of fracture. Of these, 81 (36.7%) were at high risk of hip fracture, as follows: 18 patients aged 70-80 years (17 female) and 63 patients aged 80-90 years (51 female). High risk of osteoporotic fracture was limited to two female patients (0.1%) aged over 80 years.

**Conclusion:**

FRAX^®^ analysis revealed similar fracture risks in the sample and the older adult population overall. Prospective investigation of fracture rates in home care patients, identification of true risk factors and construction of a home care patient-specific clinical score are warranted.

## RESUMO

**Objetivo:**

Avaliar o risco de fratura de quadril e fratura osteoporótica, em 10 anos, em pacientes em atenção domiciliar, de acordo com a ferramenta FRAX^®^.

**Métodos:**

Estudo transversal, retrospectivo, observacional realizados com pacientes de uma empresa de Assistência Domiciliar com idade ≥40 e ≤90 anos. Foi avaliado o risco de fratura por meio da calculadora *on-line*, tendo sido considerado elevado risco de fratura de quadril acima de 3% e elevado risco de fratura osteoporótica quando acima de 20%. Os dados foram expressos em número absoluto (n), frequência relativa (%), média, desvio padrão (±) e valor de significância (p).

**Resultados:**

Dos 222 pacientes, 83 (37,7%) apresentaram alto risco de fratura, sendo 81 (36,7%) casos por elevado risco de fratura de quadril. Destes, 18 deles tinham idade entre 70 e 80 anos (sendo 17 do sexo feminino) e 63 entre 80 e 90 anos (sendo 51 do sexo feminino). O risco elevado de fratura osteoporótica ocorreu em apenas duas pacientes do sexo feminino (0,1%), ambas com idade acima de 80 anos.

**Conclusão:**

O risco de fratura óssea verificado pela ferramenta FRAX^®^ foi semelhante na população do estudo em relação ao da população idosa em geral. A avaliação prospectiva da incidência de fraturas nos pacientes em Atenção Domiciliar, a identificação dos reais fatores de risco e a personalização do escore clínico para este grupo de pacientes se fazem necessárias.

## INTRODUCTION

Advances in health care and improved life conditions have led to a significant increase in the average life expectancy of the Brazilian population. According to the Brazilian Institute of Geography and Statistics (IBGE - *Instituto Brasileiro de Geografia e Estatística*), life expectancy in Brazil increased from 45.5 to 72.7 years between 1970 and 2008, and is expected to reach 81.29 years in 2050. Longer life expectancy has direct public health and health services management implications.^(^ ^1^ ^)^

Bone fracture is a major cause of hospitalization in elderly patients and has significant negative impacts on patient quality of life.^(^ ^2^ ^)^ Affected patients often become functionally dependent in the short- or long-run, and approximately 50% do not regain previous levels of mobility.^(^ ^2^ ^,^ ^3^ ^)^ Age-related pathophysiological changes, such as decreased bone mineral density (BMD), frequent medication use and specific socio-environmental conditions increase the risk of fracture in this population.^(^ ^4^ ^-^ ^7^ ^)^ This is therefore a relevant public health issue from the medical, social and economic standpoints.

Trauma is the leading cause of bone fracture (90%) and fractures affecting bones such as the femur are associated with high mortality rates.^(^ ^3^ ^)^ The annual incidence of hip fracture in older adults is relatively low; however, the lifetime risk of fracture amounts to 17.5% and 6% in females and males, respectively.^(^ ^8^ ^)^

Osteoporosis plays a major role in etiopathogenesis of fractures in the elderly. In the UK, 536 thousand fractures/year are related to osteoporosis, with annual costs of more than £ 4.4 billion. In the US, the estimated average annual costs amount to US$ 20 billion.^(^ ^9^ ^)^ In Brazil, average hospitalization and medication costs reached R$ 70 million in 2006 (potentially underestimated), with 35,490 deaths/year in patients aged over 60 years.^(^ ^10^ ^)^

Fracture-related mortality ranges from 5.5% to 25% (in-hospital and two years after the event, respectively).^(^ ^11^ ^)^ According to the National British Osteoporosis Guideline Group, progressive BMD loss translates into a nearly two-fold increase (per standard deviation) in fracture risk, and the predictive value of BMD for hip fracture risk is similar to the predictive value of blood pressure for stroke.^(^ ^9^ ^)^

Bone fractures have been extensively investigated in osteoporotic and non-osteoporotic women (particularly postmenopausal women), but data associating bone fracture risks and home care are scarce.^(^ ^12^ ^-^ ^15^ ^)^ However, the immobility syndrome, resulting either from previous motor issues, changes in bone metabolism or clinical conditions, is thought to be a risk factor for pathological fracture.^(^ ^16^ ^,^ ^17^ ^)^ This syndrome affects patients of various age groups, including pediatric patients, and is common in those receiving home care. Therefore, home care patients are potentially at high fracture risk.

Studies investigating hip fracture risk in home care patients revealed fracture rates of 24.4 per 1,000 individuals/year and suggested older female patients, smokers and patients with osteoporosis, gait changes, history of falls, cognitive changes and suspected undernourishment may be were at greater risk.^(^ ^14^ ^)^ Understanding fracture risks in this growing group of patients is important to support preventive, diagnostic and therapeutic measures.

## OBJETIVE

To assess the 10-year risk of hip and osteoporotic fracture in home care patients using the FRAX^®^ tool.

## METHODS

A retrospective, cross-sectional study evaluating all patients receiving home care services from a private company based in the city of São Paulo (SP). High- (home admission) and low-complexity (home care) patients aged ≥40 and ≤90 years were included. Patients with incomplete medical records were excluded.

High complexity patients are those requiring daily 12 to 24-hour nursing care, as well as multidisciplinary care (medical, physical therapy and speech therapy), often with invasive or non-invasive ventilation support. Low complexity patients are those requiring specific rehabilitation and/or care, with no need for ongoing nursing care (*e.g*., patients undergoing motor or respiratory rehabilitation, pharmacological treatment or wound care).

Data were extracted from electronic medical records (IW - Management Health System; IncoWay Copyright^©^) on May 25^th^, 2016. The FRAX^®^ scoring tool was applied using the on-line calculator (www.shef.ac.uk/FRAX); clinical data were used, but not BMD data.

Patients with hip fracture risk greater than 3% and osteoporotic fracture risk greater than 20% were defined as high-risk patients.^(^ ^18^ ^)^

Variables were expressed as percentage, or mean and standard deviation (categorical and continuous variables, respectively). Categorical variables were compared using the Fisher’s exact test. Continuous variables were analyzed using the Mann-Whitney test or confidence analysis (ANOVA) up to two and more than two groups, respectively). The level of significance was set at 5% (p<0.05).

This project (0024/2016) was approved by the Research Ethics Committee of Home Doctor, São Paulo, SP, Brazil.

## FRAX® 

The “FRAX^®^” tool is an algorithm created by the World Health Organization (WHO), in 2008, to predict the 10-year risk of hip and major osteoporotic fractures (spine, proximal humerus, hip and forearm). This tool has been validated by several international societies and organizations, and also in Brazil (in 2015).^(^ ^19^ ^,^ ^20^ ^)^

FRAX^®^ algorithms express fracture risk as percentage (%) of hip and/or major osteoporotic fractures within a ten-year period^(^ ^19^ ^-^ ^21^ ^)^ based on the following variables:^(^ ^19^ ^)^ body mass index (BMI); history of previous fractures at classical osteoporotic fracture sites; family history of hip fracture; smoking; use of glucocorticoids (prednisone ≥5mg/day or equivalent); alcoholism diagnosis of and rheumatoid arthritis (RA). Femoral neck bone density measurements may be included or not.

## RESULTS

A total of 393 patients were eligible for inclusion in the study; of these, 171 were excluded due to incomplete medical records. The final sample included 222 patients with the following characteristics: age range 41 to 90 years - 131 women and 91 men, mean age 71.5 years (±13.2 years) and 66.9 years (±14.8 years), respectively. Most patients were aged over 60 years (n=156; 70.2%) and the female gender prevailed (n=98; 62.8%). Male patients accounted for 41% of the overall sample and 38% of elderly patients. Demographic variables are shown in table 1.

**Table 1:** Demographic variables

Variable	Patients (n)	Age in years (mean±standard deviation)
Sex		
Female	131	71.5±13
Male	91	66.9±14.8
Age range, years		
40-50	27	45.5±2.9
51-60	40	55.15±2.6
61-70	35	64.6±2.9
71-80	52	75.9±2.9
>80	68	85.5±2.5
BMI		
<18,5	9	69±14.8
18.5-24.9	111	67.7±15.1
25-29.9	59	71.9±15
30-34.9	29	71.5±11.1
>35	14	71.6±11.3
Home care program		
Home care	96	70.7±14.7
Home admission	126	68.8±14.2
Total	222	69.7±١٤.٤

BMI: body mass index.

**Figure 1:**
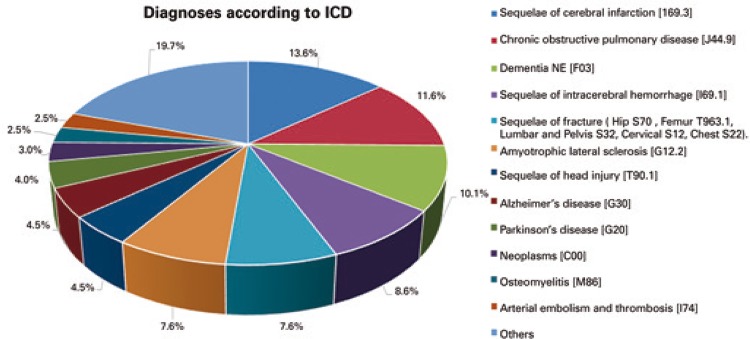
Diagnoses according to the International Statistical Classification of Diseases (ICD)

The diagnoses requiring home care (International Statistical Classification of Diseases - ICD) are listed in figure 1. FRAX^®^ analysis revealed greater average 10-year risk of bone fracture in high complexity (non-significant differences) female (p<0.001) home care patients, and direct correlations between fracture risk and advanced age in both sexes (p<0.001) (Table 2).

**Table 2:** Risk of osteoporotic and hip fracture within 10 years

	n	Osteoporotic fracture		Hip fracture	
	
		Mean and standard deviation (%)	p value	Mean and standard deviation (%)	p value
Sex					
Female	131	7.9±5.2		3.7±3.4	
Male	91	3.7±2.2	<0.001^*^	1.7±1.8	<0.0001^*^
Age group					
Adult	66	2.6±1.1		0.3±0.3	
Elderly	156	7.7±4.8	<0.001^*^	3.9±3	<0.001^*^
Age group, years					
40-50	27	2.4±1		0.2±0.3	
51-60	40	2.7±1.2		0.4±0.3	
61-70	35	3.3±1.7		1±0.9	
71-80	52	6.4±3.2		3.3±2.4	
>80	68	11.1±4.6	<0.001^†^	6±2.7	<0.001^†^
BMI					
<18.5	9	6.5±3.9		4.1 ±3.7	
18.5-24.9	111	6.5±5.2		3.2 ±3.5	
25-29.9	59	6.6±4.6		2.8 ±2.5	
30-34.9	29	5.4±3.5		2 ±1.2	
>35	14	3.9±2.7	0.2711^†^	1.2 ±1.2	0.0464^†^
Type of care					
Home care	96	5.8±4.6		2.7±2.8	
Home admission	126	6.5±4.8	0.1936^*^	3±3.2	0.3030^*^
Previous fracture and or osteoporosis					
Absent or unknown	198	5.9±4.4		2.8±2.9	
Present	24	8.6±6.5	0.0114^*^	3.6±4.2	0.3628^*^
Use of steroids^‡^					
Absent	204	6.2±4.7		2.9±3	
Present	18	6.8±4.6	0.2543^*^	3±3.7	0.7642^*^
Total	222	6.2±4.7		2.9±3	

* Mann-Whitney teste; ^†^ analysis of variance; ^‡^ steroids for over 3 month, dose of 5mg/day prednisone or equivalent.

Eighty-three (37.7%) patients in this sample were at high risk of fracture and 81 (36.7%) had hip fracture risks greater than 3%. This latter group comprised 18 patients aged between 70 and 80 years (17 women and 1 men) and 63 patients aged between 80 and 90 years (51 women and 12 men). Osteoporotic fracture risks greater than 20% were limited to two (0.1%) female patients aged over 80 years.

**Figure 2:**
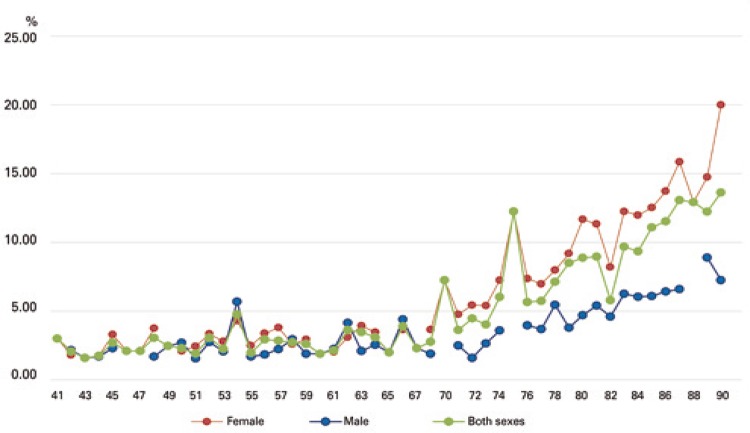
Estimated 10-year risk of osteoporotic fracture according to age and sex (FRAX® tool)

**Figure 3:**
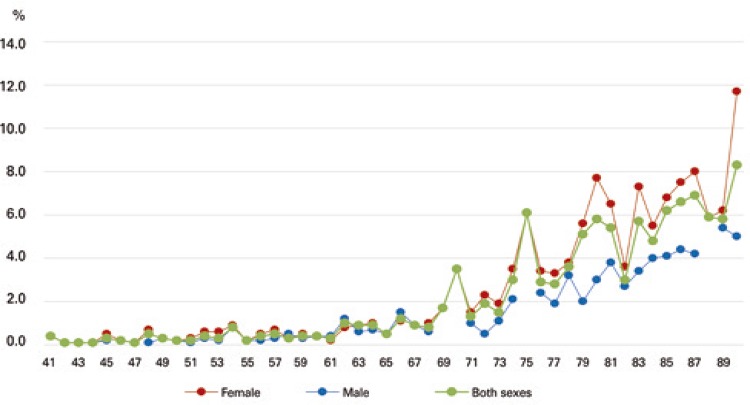
Estimated 10-year risk of hip fracture according to age and sex (FRAX® tool)

Fracture risk data according to age and sex are shown in figures 2 and 3. The 10-year risk of hip and osteoporotic fracture increased progressively with age in male and female patients alike.

## DISCUSSION

Aside from increased comorbidity burden, ageing is associated with higher risk of fall, medication use and bone fragility – and therefore with risk of fracture. Hence, home care patients (older adults for the most part) may in fact be an at-risk population.

As in previous studies, female patients (p<0.001) in this sample were at higher risk of hip and/or osteoporotic fracture, and risks increased progressively with age (p<0.001). According to pathophysiology, these findings may be explained by osteoporosis progression, muscle composition changes and environmental or behavioral factors.^(^ ^14^ ^,^ ^22^ ^-^ ^24^ ^)^

Likewirse in this study (2.86%), a Canadian cohort study with 133354 patients revealed a 2.4% hip fracture rate among elderly home care patients.^(^ ^14^ ^)^ Similar findings regarding hip and osteoporotic fracture risks were also reported in a recent US study involving 3127 individuals aged over 50 years, and evaluated using the FRAX^®^ tool (Table 3).^(^ ^18^ ^)^ To the best of the authors’ knowledge, studies describing FRAX^®^-based assessment of home care patients are lacking.

**Table 3:** Risk of hip and major osteoporotic fracture, per age group, in the present study as compared to the study by Looker et al.(^18^)

Type of fracture	Present study	Looker et al.^(^ ^18^ ^)^
Hip, years		
40-50	0.24	0.1
51-60	0.39	0.38
61-70	0.99	0.86
71-80	2.65	2.41
>80	5.6	*
Major osteoporotic fracture, years		
40-50	2.39	2.59
51-60	1.73	5.54
61-70	3.34	7.77
71-80	6.15	9.57
>80	12	11.35

* Imprecise data due to standard deviation > 50%.

Brazilian studies on the topic are scarce. In a retrospective cohort study investigating a population living in the state of Ceará (equatorial region), Castro et al., reported annual hip fracture rates of 5.59/10,000 male and 12.4/10,000 female individuals aged over 50 years – lower than figures reported by Komatsu et al., in the Southeast region of the country (12.6/10,000 male and 28.8/10,000 female individuals aged over 60 years).^(^ ^25^ ^,^ ^26^ ^)^ Continental size and ethnic heterogeneity may account for varying fracture rates in Brazil.

Individuals with Parkinson’s disease, those with cognitive impairments and undernourished patients are at greater risk for hip fracture, regardless of osteoporosis.^(^ ^15^ ^)^ Likewise, undernourished patients (BMI<18.5kg/m^2^) in this study were at greater risk for hip fracture (p=0.04). Overweight patients were at greater risk of osteoporotic fracture (non-significant differences; p=0.07).

Major conditions affecting patients in this sample involved cognitive and neurological impairments; therefore, the population considered was at high risk of fracture.^(^ ^14^ ^)^ However, it cannot be said that risks were significantly greater in this subset of patients, given multivariate analysis investigating correlations between pathological condition and fracture risk was not performed.

Chronic use of glucocorticoids is known to interfere with bone formation via inhibition of osteoblastic activity. The impacts of chronic prednisone use on bone metabolism, even at low doses, have been confirmed in a randomized trial.^(^ ^27^ ^)^ Corticotherapy was limited to 18 patients (8%) in this sample, who did not have increased fracture risks.

Home care patients are less prone to alcoholism and smoking. This was thought to be a protective factor in FRAX^®^ analysis, which may offset remaining risks in this selective patient population. Also, other potential risk factors for fracture in home care patients, such as immobility, specific underlying conditions (particularly neurological conditions), use of medications interfering with bone metabolism (*e.g*., anticonvulsant agents) and frequent physical therapy manipulation, are not accounted for in the FRAX^®^ tool. This begs the question whether application of the FRAX^®^ score as a stand-alone tool (*i.e*., independent of bone density measurements) might underestimate true fracture risks in these patients. On the other hand, the value of clinical scoring systems in scenarios where the possibility of BMD assessment is limited due transportation and/or locomotion constraints cannot be overemphasized.

Rheumatoid arthritis (RA) is the only underlying disease incorporated in the FRAX^®^ tool. RA is rarely diagnosed in home care settings and could well be replaced or complemented by more prevalent conditions associated with higher fracture risks in this subset of patients, such as cerebral palsy. Also important, clinical scores should account for patients aged under 40, including pediatric patients.

FRAX^®^ analysis of home care patients revealed similar fracture risks in this population and individuals of similar age overall. However, higher fracture rates are in fact observed in home care patients in clinical practice. Hence, deeper investigation of this specific patient subset is warranted for increased understanding of the true risk factors in this population and construction of a specific clinical risk score.

Patient exclusion due to incomplete medical records and exclusion of BMD data from the FRAX^®^ analysis were the major limitations in this study. Given BMD data were not taken into account, correlation analysis of the clinical score with and without this ancillary test was not possible.

## CONCLUSION

FRAX^®^-based fracture risk assessment in home care patients revealed increased hip and osteoporotic fracture risks with advancing age and higher risks of both fractures in female compared to male patients.

Undernourishment was significantly associated with increased hip fracture risk. Other variables accounted for in the FRAX^®^ tool were not directly correlated with fracture risk in the population studied.

Deeper investigation of fracture rates in home care patients via cohort studies may be a valuable alternative for identification of additional risk factors in this specific patient subpopulation, and may contribute to improved quality of carefor these patients.
